# How cutaneous leishmaniasis and treatment impacts in the patients’ lives: A cross-sectional study

**DOI:** 10.1371/journal.pone.0211374

**Published:** 2019-01-25

**Authors:** Endi Lanza Galvão, Mariana Junqueira Pedras, Gláucia Fernandes Cota, Ana Rabello, Taynãna César Simões

**Affiliations:** Pesquisa Clínica e Políticas Públicas em Doenças Infecto-Parasitárias–Instituto René Rachou—Fundação Oswaldo Cruz, Fiocruz, Belo Horizonte, Minas Gerais, Brazil; Institute of Tropical Medicine Antwerp, BELGIUM

## Abstract

**Background:**

Until now, few studies have evaluated the effect of cutaneous leishmaniasis (CL) on patients' quality of life, and none have used a specific instrument to measure this effect. The objective of this study was to identify factors that may be associated with the high impact of CL and to assess patients’ satisfaction with treatment and health services by utilizing a disease-specific questionnaire.

**Methodology:**

Between December 2015 and May 2017, 100 patients with localized cutaneous leishmaniasis were interviewed at a leishmaniasis referral center in Brazil. Data were collected by two questionnaires. One questionnaire compiled the sociodemographic, economic, and clinical information related to the disease. The second questionnaire was the Cutaneous Leishmaniasis Impact Questionnaire (CLIQ), which consisted of two subscales that measured 1) the general impact of CL and 2) patients’ perceptions of treatment and health services. The median scores from each of these two subscales were used to dichotomize the dependent variables. Risk factors for the high impact of CL and for low patient satisfaction with treatment and health services were analyzed with a logistic regression analysis.

**Results:**

The chance of higher impact of CL was increased in patients with the presence of comorbidities (OR: 3.9; CI 1.25–12.36), in those with absences from work (OR: 12.0; CI 3.78–42.55), in those who relied on public transportation by a municipal bus (OR: 5.8; CI 1.27–26.77), and in those who had illness-related expenses greater than U$137 (OR: 3.5; CI 1.17–10.24). The chance of patient dissatisfaction with treatment and health services increased with higher education (OR: 5.0; CI 1.19–21.03) and with illness-related expenses exceeding U$137 (OR: 4.64; CI 1.49–14.48). Once the sample was non-probabilistic, findings are not representative of CL patients in general.

**Conclusions:**

CL and its treatment have a negative impact on patients’ quality of life. Considering these effects during public health planning may help patients to confront the disease.

## Introduction

Currently, with changes in the biomedical paradigm, it is recognized that an individual's subjective experiences should be included as indicators when evaluating the traditional epidemiology of diseases so as to improve health outcomes. In the last few decades, health status self-assessment has been a widely used indicator [1–7]. Individuals’ perceptions about their own health are usually assessed by asking a single question: *“How do you rate your health*: *very good*, *fairly good*, *average*, *fairly poor*, *or poor*?*”*. However, a deeper understanding of a particular health condition and of the impact of a disease contributes to guiding policy decisions considering the specific needs of the population.

In Brazil, the health needs of the population are provided for by the public health system, which addresses health inequalities and offers “health for all”. In this context, the Unified Health System (SUS) is a free at point of use and offers health promotion, diagnosis, medication, ambulatory care and hospitalization for the Brazilian population [8].

Cutaneous leishmaniasis (CL) represents a public health problem in the Americas, especially in Brazil, affecting the poorest people who have the greatest difficulty in accessing health services [9]. It is a neglected tropical disease and the most common form of leishmaniasis. In the localized form, the disease only affects the skin and is nonfatal, but patients may have multiple lesions and are in more pain than they may appear. Moreover, the associated chronic ulcerative scars can cause not only permanent disfiguration that leads to psychological disorders [10,11] but also social prejudice [12] and permanent changes in individuals’ perceptions of self [13].

Studies have demonstrated a moderate to large negative effect of CL on the quality of life (QoL) of patients [12,14]. The most affected area of QoL was “symptoms and feelings” [14,15], while the least affected areas were “personal relationships” [12], “sexual function” [15], and “treatment” [14]. None of these studies investigated factors that could be related to these effects. Usually, the impact of CL is associated only with the presence of active lesions [16,17]. However, there is a possibility that sociodemographic and economic factors and treatment conditions may influence the way that people perceive the disease. Few studies have investigated the impact of CL on patients, and they have only evaluated these characteristics through a nonspecific CL questionnaire [10,12,14,15].

Therefore, the aim of this study was to specifically identify factors that may be related to the impact of CL on the QoL of Brazilian adult patients and to evaluate their perceptions of treatments for leishmaniasis and health services with a specific tool developed for assessing the QoL of CL patients.

## Materials and methods

### Study design, inclusion criteria and settings

The present cross-sectional study was conducted at a referral center for leishmaniasis [*Centro de Referência em Leishmanioses do Instituto René Rachou*, *Fundação Oswaldo Cruz*] in Belo Horizonte, Minas Gerais, Brazil. From December 2015 to May 2017, one hundred consecutive outpatients compounded this convenience sample. All selected patients had cutaneous leishmaniasis. parasitologically confirmed. The purpose of the study was explained to the patients, and signed informed consent was obtained from each participant. The study was approved by the Ethics Committee on Human Research of the René Rachou Institute [*Fundação Oswaldo Cruz*, *n°* 1.337.731, approval on November 25, 2015]. The population sample studied here was the same reported in a broader study addressing the development and validation of the Cutaneous Leishmaniasis Impact Questionnaire (CLIQ) [18].

The inclusion criteria were as follows: age greater than 18 years and treatment initiated within at least five days and at most 90 days. Only patients with the localized form of cutaneous leishmaniasis were included. Patients with non-CL-related wounds and cognitive problems that hindered comprehension of the scale were excluded.

### Study variables and data collection

This study sought to answer two main questions: a) What factors influence the increase in the impact of CL as perceived by affected individuals? b) What factors influence patient satisfaction with treatment and with health services in the context of this disease?

Data were collected using two questionnaires. The first one was the Cutaneous Leishmaniasis Impact Questionnaire (CLIQ), a questionnaire that was previously developed and validated for Brazilian patients [18] and that includes questions regarding the perception of patients about factors related to CL and about their satisfaction with treatment and with health services. This questionnaire was developed following steps based on literature review, a *panel of experts*, an exploratory interview with CL patients and a pilot study with psychometrics analysis. One of the main limitations observed during the validation process was linked to the relatively small sample and to the little variability of data, once all patients were from the same referral center. The other questionnaire included sociodemographic and economic data and clinical features. The questionnaires were administered by a single trained researcher to all patients who agreed to participate in the survey. A pilot study using the two questionnaires was performed before the start of the interviews to help identify any unexpected problems that might arise during the study, thus reducing the amount of missing data. Another single researcher performed the transcription of data from paper forms into an electronic database.

Two outcome variables were used to evaluate patients’ perceptions of the impact of CL on QoL. To collect this information, the CLIQ was used; this questionnaire consisted of 25 questions distributed into two subscales: 1) the general impact of CL and 2) patients’ perceptions of treatment and health services. In this questionnaire, the final score was obtained by adding the numerical response codes of all the items. The scores could range from 0 to 72 and from 0 to 28 points, respectively, for each subscale. A higher score denoted a greater negative perception of patients regarding their QoL. The analysis was carried out using the median of each subscale as the cut-off point. Therefore, in terms of the subscale that assessed the general impact of CL, the outcomes were dichotomized as “low impact” and “high impact” groups. In terms of the subscale that assessed patients’ perceptions of treatment and health services, the outcomes were dichotomized as “low satisfaction” and “high satisfaction” groups.

The independent variables affecting QoL were related to sociodemographic, economic and clinical status, in addition to characteristics associated with patients’ access to health services. These data were assessed through another questionnaire that was piloted prior to the data collection. The dependent and independent variables were used in the regression model.

The sociodemographic variables corresponded to gender, age, marital status, educational status, family income and per capita income. Age and income were analyzed in a continuous form. Marital status was reported as “married” or “unmarried”. Educational status was reported in three categories: “higher education or more”, “up to secondary education”, and “up to primary education”. Some variables were considered in the evaluation of economic factors: absenteeism and the number of absences from work. In addition, the total cost of the disease for the patients was analyzed according to the following categories: “up to 137 dollars” and “more than 137 dollars” (1 American dollar = 3.28 BRL). Patients’ need to spend financial resources as a result of CL was assessed in a dichotomous manner in relation to the following expenses: medical consultations, hospitalization, medical examinations, medications, transportation, out-of-home meals, childcare, domestic and labor services, bandage materials and health insurance.

The following were clinical characteristics of interest: the number, size and location of lesions; the presence of ulcerated lesions, lymphadenopathy, lymphangitis and secondary infections; the treatment option in progress the length of treatment; illness time; recurrence; and the time between diagnosis and initiation of treatment. These data were collected from the patients' records. Furthermore, participants were asked about the presence of comorbidities and the need for hospitalization related to CL.

In addition, other variables related to patients’ access to health services were collected. In these instances, the distance traveled for CL diagnosis and treatment was evaluated according to the following categories: "up to 30 km", "between 30.1 and 100 km", and "over 100 km". The type of health service where the patient was diagnosed was recorded as "public" or "private". The number of appointments required for diagnosis was recorded as "one", "two to four", or "five or more". The mode of transportation used by patients to travel to the CL treatment site and the use of private health insurance were also variables of interest.

Patients’ adherence to therapy was evaluated according to the modified Morisky test [19], which included the following three questions: 1) Have you ever forgotten to take your medicine? 2) When you feel better, do you sometimes stop taking your medicine? 3) At times, if you feel worse when you take your medicine, do you stop taking it?

### Statistical analysis

Initially, histograms and the Kolmogorov-Smirnov test were used for distribution analysis. Descriptive statistics were used to describe the sociodemographic and economic data and clinical features. Percentages and frequencies were used for the categorical independent variables, while the means and standard deviations were calculated for the continuous independent variables. The median was used as the cut-off point for analysis of the outcomes of interest to dichotomize the scores of each of the two CLIQ subscales (1- general impact of CL and 2- perceptions of treatment and health services). The logistic regression analysis followed six steps suggested by Hosmer & Lemeshow [20]. First, univariate logistic regression models were used to identify the association between the variables. The variables with a p-value less than 20% in this test were included in the multivariate logistic regression analysis. The follow five steps were related to the inclusion and exclusion testing of variables to complete the final model. Before completing the modeling, each of the interactions between the variables in the model were tested. Collinearity statistics were also used to assess possible collinearity between covariates. The odds ratios (ORs) and 95% confidence intervals (CIs) for the “low impact” and “high impact” groups and for the “low satisfaction” and high satisfaction” groups were calculated. Data were analyzed using R software version 3.4.0 (The R Foundation for Statistical Computing http://www.r-project.org/), with the lmtest and caret packages.

## Results

### Characteristics of the patients and disease

A total of 71 male (71%) and 29 female (29%) patients between 19 and 81 years of age (mean 44.96 years) were enrolled in the present study. Among them, 57% were married. Regarding educational level, 54 (54%) had completed primary education, 27 (27%) had completed secondary education, and 19 (19%) had higher college education or more. The family income and per capita income for 50% of patients were U$759.00 (range: U$0—U$4878.04) and U$253.00 (range: U$0—U$1524.39), respectively. Two patients reported that they were unemployed with no income at the time of data collection.

The duration of the lesions varied from four weeks to two years (mean of 3.6 months and median of 3 months). Most patients had a single lesion (69%), while 14% had two lesions, and 17% had three to five lesions. In terms of the lesion location, 10.6% were located on the trunk, 17.5% on the neck or face, 35% on the lower limbs, and 36.9% on the upper limbs. The most frequent clinical presentation was ulcerated lesions (83%). The size of the lesions ranged from 0.3 cm to 11.7 cm in diameter. During the period of the study, a trial was under way in the referral center, justifying that about half of the patients were treated with intralesional infiltration approach. In terms of treatment, 51% of patients were treated with meglumine antimoniate intralesional infiltration, and 49% were treated with intravenous meglumine antimoniate. We observed 100% adherence to treatment in both drug groups. Thirty-seven percent of patients reported comorbidities, and the most common concomitant disease was systemic arterial hypertension (45.9%), followed by depression (8.1%).

Only 11% of patients received a diagnosis of CL during their first visit to a healthcare provider, and 90% of diagnostic confirmation was performed by a public health service. A total of 69 (69%) subjects traveled a distance of up to 100 km in order to obtain diagnostic confirmation of CL. In terms of the distance traveled to initiate treatment, 58% of patients had to travel up to 30 km. In addition, 56% of patients needed to miss work (or school) at some time because of CL-related treatment or consultations. The most commonly used mode of transportation was the patients’ own cars (40%), followed by public health transportation (24%). Overall, 38% of patients had private health insurance, which was used by ten patients (73.6%).

### CLIQ scores

The mean CLIQ score was 21.45 (± 14.03) for the general impact of CL subscale and 6.48 (± 4.07) for the perception of treatment and health services subscale, which included a total of 72 and 28 points, respectively. These subscales were divided into medians to represent low and high scores to study frequencies. Thus, the medians for the general impact of CL subscale and the perception of treatment and health services subscale were 18.5 and 6.0 points, respectively. No patient scored zero on the general impact of CL subscale, indicating that all patients reported some negative impact of the disease. In the perception of treatment and health services subscale, 4% of patients scored a total of zero points, indicating complete satisfaction.

### Univariate analysis

In the univariate analysis, the presence of comorbidities (p = 0.002), expenses related to bandage materials (p = 0.006), absenteeism (p = 0.016) and the number of absences (p < 0.001) were significantly associated with the negative impact of the disease. In addition, expenses related to medical consultations (p = 0.022) and CL medications (p = 0.016) and expenses greater than U$137 dollars (p = 0.010) were also associated with high impact.

In terms of patient satisfaction with treatment and health services, the following variables were significant in the univariate analysis: educational status (p = 0.042), distance traveled to obtain the diagnosis of the disease (p = 0.011), number of times that patients needed to travel for CL treatment (p = 0.020), use of public transportation to access health services (p = 0.039), medication side effects (p = 0.035), presence of lymphadenopathy (p = 0.020), presence of lymphangitis (p < 0.001), expenses related to bandage materials (p = 0.008) and expenses exceeding U$137 dollars (p = 0.003). Table 1 summarizes the associations between socioeconomic, clinical and related variables and variables related to access to health services and the general impact of CL and patients’ satisfaction with treatment and health services.

**Table 1:** Univariate analysis of the general impact of CL subscale and the perceptions of treatment and health services subscale according to sociodemographic, economic and clinical variables and variables related to access to health services.

	General impact of CL (n = 100)				Perceptions of treatment and health services (n = 100)			
Characteristics	Low impact	High impact	OR (95% CI)	p-value	High satisfaction	Low satisfaction	OR (95% CI)	p-value
**Age**	-	-	1.01 (0.99–1.04)	0.125*	-	-	1.01 (0.99–1.04)	0.207
**Gender**								
Female	12	17	1		18	11	1	
Male	38	33	0.61 (0.25–1.46)	0.272	38	33	1.42 (0.58–3.43)	0.436
**Highest education level completed**								
Primary school or lower	27	27	1		32	22	1	
Secondary school	14	13	0.92 (0.36–2.34)	0.875	18	9	0.72 (0.27–1.91)	0.518
Higher education	9	10	1.11 (0.39–3.16)	0.844	6	13	3.15 (1.03–9.55)	0.042 *
**Family income**	-	-	0.99 (0.99–1.00)	0.271	-	-	1.00 (0.99–1.00)	0.353
**Per capita income**	-	-	0.99 (0.99–1.00)	0.464	-	-	1.00 (0.01–54.83)	0.627
**Health insurance**								
No	30	32	1		36	26	1	
Yes	20	18	0.84 (0.37–1.89)	0.680	20	18	1.24 (0.55–2.80)	0.595
**Health service where the diagnosis was confirmed**								
Public	46	44	0.63 (0.16–2.41)	0.508	48	42	3.50 (0.70–17.40)	0.125*
Private	4	6	1		8	2	1	
**Comorbidity**								
No	39	24	1		37	26	1	
Yes	11	26	3.84 (1.61–9.16)	0.002*	19	18	1.34 (0.59–3.05)	0.473
**Number of lesions**								
One lesion	35	34	1		41	28	1	
Two or more lesions	15	16	1.09 (0.47–2.56)	0.829	15	16	1.56 (0.66–3.66)	0.304
**Lesion size**	-	-	0.99 (0.99–1.00)	0.733	-	-	1.00 (0.08–11.84)	0.801
**Location of lesions**								
**Lower limb**								
No	31	25	1		23	23	1	
Yes	19	25	1.63 (0.73–3.61)	0.228	23	21	1.16 (0.74–1.80)	0.504
**Upper limb**								
No	26	31	1		32	25	1	
Yes	24	19	0.81 (0.53–1.22)	0.322	24	19	1.30 (0.59–2.90)	0.506
Lesion appearance **								
Ulcerative	42	41	1		48	35	1	
Non-ulcerative	8	8	1.02 (0.35–2.98)	0.965	8	8	1.37 (0.46–4.00)	0.564
Presence of secondary infection **								
No	44	44	1		49	39	1	
Yes	5	4	0.80 (0.20–3.17)	0.751	6	3	0.62 (0.14–2.67)	0.529
Lymphangitis **								
No	46	45	1		48	43	1	
Yes	3	4	1.36 (0.28–6.43)	0.696	7	0	0.62 (0.14–2.67)	<0.001*
Lymphadenopathy **								
No	42	43	1		43	42	1	
Yes	7	6	0.83 (0.25–2.69)	0.766	12	1	0.08 (0.01–0.68)	0.020*
Onset of injury **								
Up to 90 days	32	34	1		35	31	1	
More than 90 days	18	15	0.78 (0.33–1.81)	0.570	20	13	0.73 (0.31–1.71)	0.475
**Relapse after cure**								
No	47	49	1		53	43	1	
Yes	3	1	0.31 (0.03–3.18)	0.331	3	1	0.41 (0.04–4.09)	0.448
**Intravenous meglumine antimoniate**								
No	27	24	1		25	26	1	
Yes	23	26	1.27 (0.57–2.78)	0.549	31	18	0.55 (0.25–1.24)	0.153*
**Intralesional treatment time**								
Up to 2 days	39	37	1		46	10	1	
More than 2 days	11	13	1.24 (0.49–3.12)	0.640	30	14	2.14 (0.84–5.45)	0.108*
**Intravenous treatment time**								
Up to a week	32	29	1		32	29	1	
More than a week	18	21	1.28 (0.57–2.88)	0.539	24	15	0.68 (0.30–1.56)	0.373
**Side effects**								
No	30	24	1		25	29	1	
Yes	20	26	1.62 (0.73–3.58)	0.230	31	15	0.41 (0.18–0.94)	0.035*
**Need for hospitalization**								
No	48	43	1		53	38	1	
Yes	2	7	3.90 (0.76–19.83)	0.100*	3	6	2.78 (0.65–11.85)	0.165*
Absenteeism **								
No	23	33	1		23	11	1	
Yes	23	11	0.33 (0.13–0.81)	0.016*	29	27	1.94 (0.79–4.73)	0.142*
**Number of absences from work**								
Until 6	40	10	1		33	24	1	
More than 6	17	33	7.76 (3.13–19.23)	<0.001*	23	20	1.19 (0.53–2.65)	0.660
**Distance traveled for diagnosis**								
Up to 30 km	22	13	1		15	20	1	
30.1 to 100 km	14	20	2.41 (0.91–6.36)	0.073*	18	16	0.66 (0.25–1.72)	0.402
More than 100 km	14	17	2.05 (0.76–5.50)	0.151*	23	8	0.26 (0.09–0.74)	0.011*
**Distance traveled for treatment**								
Up to 30 km	31	27	1		29	29	1	
30.1 to 100 km	11	10	1.04 (0.38–2.83)	0.933	14	7	0.50 (0.17–1.41)	0.193*
More than 100 km	8	13	1.86 (0.67–5.17)	0.231	13	8	0.61 (0.22–1.70)	0.351
Number of consultations until diagnostic confirmation **								
One	6	2	0.28 (0.04–1.58)	0.151*	4	4	0.94 (0.20–4.38)	0.942
Two to four	27	29	0.90 (0.38–2.10)	0.816	34	22	0.61 (0.26–1.43)	0.258
Five or more	16	19	1		17	18	1	
Need of health services for routine consultation **								
Up to five consultations	29	23	0.54 (0.24–1.23)		32	20	0.57 (0.25–1.28)	0.177*
Six or more	18	26	1	0.148*	21	23	1	
Need of health services for treatment **								
Up to seven consultations	29	31	1.12 (0.50–2.52)	0.774	39	21	0.37 (0.16–0.85)	0.020*
Eight or more	20	19	1		16	23	1	
**Expenses with:**								
**Medical consultations**								
No	46	37	1		47	36	1	
Yes	4	13	4.04 (1.21–13.43)	0.022*	9	8	1.16 (0.40–3.30)	0.780
**Medical examinations**								
No	36	35	1		41	30	1	
Yes	14	15	1.10 (0.46–2.61)	0.826	15	14	1.27 (0.53–3.03)	0.583
**Medications**								
No	35	23	1		33	25	1	
Yes	15	27	2.73 (1.20–6.22)	0.016*	23	19	1.09 (0.49–2.42)	0.832
**Transportation**								
No	36	36	1		36	36	1	
Yes	14	14	1.00 (0.41–2.39)	1	20	8	0.39 (0.15–1.02)	0.056*
**Meals outside the home**								
No	22	13	1		21	14	1	
Yes	28	37	0.44 (0.19–1.30)	0.061*	35	30	0.77 (0.33–1.79)	0.555
**Bandage materials**								
No	34	20	1		37	17	1	
Yes	16	30	1.76 (1.17–2.64)	0.006*	19	27	1.86 (1.17–2.95)	0.008*
**Health insurance**								
No	41	37	1		46	32	1	
Yes	9	13	1.60 (0.61–4.17)	0.336	10	12	1.72 (0.66–4.47)	0.262
Total expenses **								
Up to 137 dollars	28	16	1		31	13	1	
More than 137 dollars	15	27	3.14 (1.30–7.60)	0.010*	16	26	3.87 (1.57–9.51)	0.003*
**Means of transport**								
**Own car**								
Yes	22	18	0.71 (0.32–1.59)	0.415	23	17	0.90 (0.40–2.02)	0.805
No	28	32	1		33	27	1	
**On foot**								
Yes	10	7	0.65 (0.22–1.87)	0.427	9	8	1.16 (0.40–3.30)	0.780
No	40	43	1		47	36	1	
**Public transport by municipal bus**								
Yes	6	11	2.06 (0.69–6.11)	0.189*	10	7	0.87 (0.30–2.50)	0.797
No	44	39	1		46	37	1	
**Sanitary bus**								
Yes	11	13	1.24 (0.49–3.12)	0.640	9	15	2.70 (1.04–6.96)	0.039*
No	39	37	1		47	29	1	

OR: odds ratio; 95% CI: 95% confidence interval

* p < 0.2

** Totals vary due to missing data

### Multivariate analysis

The multivariate analysis showed that people with comorbidities have a greater chance of having higher scores on the subscale related to the impact of CL than those who did not have comorbidities (OR: 3.9 95% CI: 1.25–12.36). In addition, missing work for more than six days was associated with high impact of the disease (OR: 12.0 95% CI: 3.78–42.55). Patients who traveled by public transportation via municipal bus for disease assistance had high scores on the subscale and had more chance of high impact of disease than those who did not travel by this means (OR: 5.8 95% CI: 1.27–26.77). Finally, illness-related expenses that were greater than U$137 dollars also increased the chance of high impact of CL (OR: 3.5 95% CI: 1.17–10.24) (Table 2).

**Table 2:** Multivariate analysis showing the association between the high impact of CL and sociodemographic, economic and clinical variables and factors related to access to health services.

Variables	Unadjusted OR	95% CI	p-value	Adjusted OR	95% CI	p-value
**Number of absences from work**						
Up to six	1			1		
More than six	7.76	3.13–19.23	0.016*	12.0	3.78–42.55	<0.001*
**Comorbidity**						
No	1			1		
Yes	3.84	1.61–9.16	0.002*	3.9	1.25–12.36	0.019*
**Expenses**						
Expenses up to U$137	1			1		
Expenses greater than U$137	3.14	1.30–7.60	0.010*	3.5	1.17–10.24	0.024*
**Public transport by municipal bus**						
No	1			1		
Yes	2.06	0.69–6.11	0.189	5.8	1.27–26.77	0.023*

OR: odds ratio; 95% CI: 95% confidence interval

* p < 0.05

Similarly, patients’ satisfaction with treatment and health services was statistically associated with illness-related expenses. Thus, those who spent over U$137 dollars had more chance of being dissatisfied with treatment and health services than those who spent less than this amount (OR: 4.64 95% CI: 1.49–14.48). In this case, patients with higher education were more likely to have low satisfaction with treatment and health services than those who did not have this training (OR: 5.0 95% CI: 1.19–21.03) (Table 3).

**Table 3:** Multivariate analysis showing the association between low satisfaction with treatment and health services and sociodemographic, economic and clinical variables and factors related to access to health services.

Variables	Unadjusted OR	95% CI	p-value	Adjusted OR	95% CI	p-value
**Levels of education**						
Primary school	1			1		-
Secondary school	0.72	0.27–1.91	0.518	0.58	015–2.23	0.436*
College or higher	3.15	1.03–9.55	0.042*	5.0	1.19–21.03	0.027*
**Absences from work**						
No	1			1		
Yes	1.94	0.79–4.73	0.142	2.79	0.82–9.49	0.100
**Expenses**						
Expenses up to U$137	1			1		
Expenses greater than U$137	3.87	1.57–9.51	0.003*	4.64	1.49–14.48	0.008*
**Sanitary bus**						
No	1			1		
Yes	2.70	1.04–6.96	0.039*	3.47	0.97–14.35	0.055

OR: odds ratio; 95% CI: 95% confidence interval

* p < 0.05

## Discussion

The burden of CL on individuals has often been attributed to the physical disfigurement caused by the disease [17]. However, broadening the focus of the problem by raising issues that extend beyond physical impairment and changes in patients' appearance can refine the understanding of the disease as a public health problem and allow better allocation of public investments. In the present study, among the variables related to the clinical manifestations of CL, none of them were significantly associated with the impact of the disease in the univariate analysis. On the other hand, our study revealed that the presence of comorbidities, missing work, relying on public transportation by municipal bus and illness-related expenses greater than U$137 dollars were associated with high impact of CL on patients' QoL. In the same sense, illness-related expenses greater than U$137 dollars and higher education status were factors associated with patients’ dissatisfaction with CL treatment and health services.

In Brazil, CL diagnosis and treatment are available through the public health system, which covers the direct costs associated with the disease. Although this health system has comprehensive coverage and is based on the principles of universal access, most patients needed to spend additional money for expenses related to the disease. Moreover, illness-related expenses greater than U$137 dollars significantly impacted the quality of life of patients included in this study and influenced their perceptions regarding treatment and health services. The minimum monthly wage in Brazil in 2016 and 2017 at the time of data collection was U$268.29 and U$285.67, respectively. Thus, considering patients’ low income and that CL affects vulnerable populations [21], the government should consider new strategies for managing CL to minimize the expenses of patients with the disease. Greater investments in structuring (physical and human resources) decentralized health services would be useful to provide the correct diagnosis and meet the demands of CL patients. This strategy would reduce the number of unnecessary consultations and incorrect medications prescribed, reducing the costs of patients with the disease and the associated negative effects. Studies focusing on the detailed costs from the perspective of CL patients are necessary to guide public policies and resource allocation.

Many are the barriers that increase vulnerability and the risk for treatment non-adherence, specially lack of social incentives at municipal level [22]. In Brazil, the payment for the public transport is provided by the patients or their family. In this way, part of the expenses are related to the displacement to the health service [23]. Our results suggested that patients who depended on public transportation by municipal buses were more affected by the disease than those who do not depend on this mode of transportation. The dissatisfaction of CL patients with various aspects related to the environmental domain, such as the availability of financial resources, freedom, safety, availability and quality of transportation, has already been reported in a study conducted in Tunisia [11]. Syed et al. (2013) suggested that a lack of or inaccessibility to transportation may be associated with lower utilization of healthcare services, lack of regular medical care, and missed medical appointments, especially for low-income populations [24]. Although the type of transportation used influenced patients’ perceptions of the impact of CL, it did not influence their adherence to treatment in our study. However, future studies exploring the causes (vehicle access, travel time, cost, transportation safety) that make transportation a barrier for CL patients may help to guide future interventions.

It is already well established in the literature that HIV co-infection has the potential to negatively affect the course of CL, promoting more severe clinical presentations and poor treatment responses [25]. In the present study, none of the patients reported having HIV. Nevertheless, the presence of comorbidities was a preponderant factor that affected patients’ perceptions of the impact of CL. Consistent with the literature, the increase in the number of medical comorbidities was related to a worse perception of health status in Brazilian populations [1,3]. The most prevalent disease reported by CL patients in this study was systemic arterial hypertension (45.9%), which was also reported to be the most prevalent disease among patients with mucosal leishmaniasis (43%) in another study conducted in Brazil [26]. As the presence of comorbidities was a variable that was collected directly from the patient, it is possible that there was a tendency to underreport diseases.

Absenteeism was also a variable that negatively influenced the QoL in CL patients. In the same sense, in a study conducted by Toledo Jr (2013) in Brazil, the domain "work and school" was most frequently compromised by the occurrence of this disease [12]. The therapeutic options for CL in the Americas are long-term medications that require administration by healthcare professionals [27]; therefore, it must be concluded that work-related absences may be related to the demand for treatments and not only to the clinical manifestation of the disease. In the present study, two therapeutic options were used for patients: meglumine antimoniate administered via intralesional or intravenous routes. Intravenous treatment requires daily injections for 20 to 30 days and is associated with several side effects, which makes this option contraindicated for patients with cardiac or renal disease. Intralesional infiltration requires a more flexible schedule and lower total doses of antimoniate; thus, it has less toxic effects [28]. For this reason, we expected that the intravenous meglumine antimoniate was related to the greater impact of the disease and the low satisfaction with the treatment and health services. However, we did not identify this relationship, probably as a result of the patients being very satisfied with the treatment in general and with the care provided by a referral center for the disease.

CL causes substantial suffering due to skin scabs and the resulting social stigma [29,30]. Some qualitative studies including patients from Morocco and Tunisia reported that women suffered the greatest impact from CL compared with men mainly because the disease affected beauty and appearance [11,29]. In this study, we did not differentiate the impact of the disease according to the sex of the patients, similar to a study including Turkish people [10]. Ranawaka (2014) reported that the quality of life of men was more affected by disease than that of women in Sri Lanka [15]. Thus, although men are more affected by CL than women, there is a lack of data demonstrating the presence or not of a differential impact of the disease quality of life according to gender. Our findings should be interpreted with caution since it is a small sample size and the small number of women assessed may have influenced our results. Further studies with longer follow-up, larger samples and using different approaches, such as the qualitative methodology could add information to these presented here.

In general, the CLIQ scores were low (representing that the impact of the disease was perceived to be low), which shifted the medians from the subscales to below their respective midpoints. However, we considered that only a total score equal to zero was indicative of no impact of the disease, and any value above zero indicated some negative effect of CL. For this reason, we chose to classify the effects as high/low impact and low/high satisfaction. In contrast, studies evaluating the impact of CL with the Dermatology Life Quality Index (DLQI) [31] used a rating by dividing the scale into five categories, namely, “no effect”, “small effect”, “moderate effect”, “very great effect” and “extremely great effect”; in these studies, patients who scored from zero to 1 were classified as no effect. The reason for this categorization is not completely clear in the validation study of this instrument [31]. Using this questionnaire, 27% of the Iranian patients evaluated by Vares et al. (2013) [14] and 21% of the Sri Lankan patients evaluated by Ranawaka (2014) [15] reported no effect of CL on their quality of life surveys. A previous study using DLQI [12], which was conducted in the same outpatient clinic as the present study, corroborated our findings that all patients had some negative effects related to the disease.

When we evaluated the subscale “perceptions of treatment and health services”, medication side effects were significant in the univariate model but were not significant in the final model. The same phenomenon occurred when considering the type of medication used by the patient (intravenous antimoniate), which is closely related to the presence of side effects [28]. Thus, although the toxicity of CL treatment is a reality and new therapeutic alternatives are necessary for those with contraindications for available drugs, this was not an issue that impacted patients in this study. Patients’ perceptions of improvement in their lesions evolving towards a cure seemed to have greater relevance than the side effects of the medication [32].

A study conducted in Brazil reported that dissatisfaction with the public health system increased by 7% for each unit in the literacy rate [33]. Our study revealed that having college education increased patients’ dissatisfaction with CL treatment and health services by 5 times, confirming that more educated people were more demanding about health care.

In general, scores on the subscale “perceptions of treatment and health services” were low, representing high satisfaction with respect to these aspects. It should be noted that the interviews were conducted at a leishmaniasis referral center and that the demands for treatment had already been resolved. According to the literature, an unresolved demand increases the odds of dissatisfaction with the Unified Health System in Brazil when compared with resolved demands [33].

The extent of psychosocial suffering from CL patients was recently summarized by Bennis *et al* [34] that identified relevant committed aspects: anxiety and depression, low quality of life, fear of scars, expectation of stigmatization, perception of changed body image, social stigma, self-stigma and resistance to stigma. In addition, Al-Kamel [35], in a qualitative study, reveled CL-related social isolation, aesthetic impairment and emotional stress in Yemen patients. The present study provides an understanding of the effects that CL may have on the patients regarding sociodemographic, economic and clinical status, taking a step beyond the findings already established. Our results are relevant for planning strategies to manage this disease. Studies that aim to assess the perceptions of CL patients being treated with different therapeutic interventions may point to valuable ideas on how to improve patients' quality of life from a treatment perspective [11].

The main limitation of this study is its reverse causality bias, which is inherent in cross-sectional studies. Thus, although the statistical model simulates a model of causal determination, it is not possible to assure the order of occurrence of the variables. Another limitation is that the analyses were performed using the cut-off points of the questionnaire, which were the medians of each subscale. Future studies should analyze different cut-off points to discriminate the greater or lesser impact of CL on patients' quality of life as well as on their satisfaction with treatment and health services, especially considering diverse populations. The lack of sample size calculation and the common origin of the patients are factors that limit the extrapolation of these results.

Despite its limitations, this study was conducted using a CL questionnaire, which has not been performed to date for this disease and showed that CL and CL treatment have a negative impact on patients' QoL. Healthcare professionals should pay special attention to patients’ comorbidities to control the symptoms and exacerbations of disease and to reduce the negative effects of CL and its treatment. Challenges related to patients’ mode of transportation and illness-related expenses beyond those subsidized by the public health system deserve government efforts to attempt to render these services more efficient and affordable. Finally, improving access to the correct diagnosis of CL and investing in research on new therapeutic alternatives that require less patient travel may reduce patients’ work absences and improve their QoL. Further studies need to be developed to explore these issues in different socio and cultural contexts.

## Supporting information

S1 File Bank of data set. (XLSX)
